# Dataset of lipids, antioxidative status and color attributes in cows meat from slaughter to storage: Impacts of diet supplementations and pre-slaughter stress

**DOI:** 10.1016/j.dib.2020.105409

**Published:** 2020-03-16

**Authors:** Mylène Delosière, Agnès Thomas, Claudia E.M. Terlouw, Dominique Gruffat, Mihaela Habeanu, Denis Durand

**Affiliations:** a INRAE, Université Clermont Auvergne, VetAgro Sup, UMR Herbivores, F-63122 Saint-Genès-Champanelle, France; b National Research-Development Institute for Biology and Animal Nutrition, Balotesti, Romania

**Keywords:** Cows, Diet, Stress, Meat, Packaging, Lipid, Oxidation, Color

## Abstract

This data article presents a dataset with 34 values of the fatty acids composition and of indicators of lipid oxidation determined in the *Longissimus dorsi* and *Semitendinosus* from 71 Normand cull-cows at slaughter, after muscle aging and after meat storage periods under different packaging conditions. Cows were subjected to 3 feeding diets and 2 slaughter protocols relative to pre-slaughter stress. The indicators of lipids, FA composition, antioxidative enzymes activities, antioxidative status and global lipid oxidation of the muscles, and meat at different time points and under different aging and storage conditions, may be used to increase our understanding of the evolution of oxidation and consequences on color development. The last research article published on part of these data [1] is available for some interpretive insights: https://doi.org/10.1016/j.foodchem.2019.125668.

Specifications table

**Table:** 

Subject	Biochemistry, biology
Specific subject area	Cows, meat, lipid, oxidation, color
Type of data	Tables, figures
How data was acquired	Gas and liquid chromatography, spectrophotometry (precisions in Table 1)
Data format	Raw and analyzed
Parameters for data collection	During the breeding period, data were collected to study the effects of the animal diet supplementation and the pre-slaughter stress. After slaughter, data were collected to study the effects of muscle aging and meat storage under different packaging conditions.
Description of data collection	The muscle and meat samples collected in refrigerated (+4 °C) conditions were immediately frozen in liquid nitrogen in order to avoid lipid oxidation due to sampling. The indicators of lipids, antioxidative status and color attributes of muscle and meat were collected after biochemical assays using published methods.
Data source location	INRA, Theix, St-Genès-Champanelle, France
Data accessibility	Dataset is available in public repository: Portail Data INRAE (data.inrae.fr) Data identification number: doi:10.15454/T6AMBC https://doi.org/10.15454/T6AMBC During the reviewing process by Data In Brief, please find data in this private URL: https://data.inra.fr/privateurl.xhtml?token=bb640bf1-37ce-4a85-9c0f-770cd9cc8b09
Related research article	[2] Gobert, M., Gruffat, D., Habeanu, M., Parafita, E., Bauchart, D. & Durand, D., Plant extracts combined with vitamin E in PUFA-rich diets of cull cows protect processed beef against lipid oxidation. Meat Science. 85 (2010) 676–683. https://doi.org/10.1016/j.meatsci.2010.03.024.

## Value of the Data

• This dataset is useful for ruminant researchers to provide an overall view of the global lipid content and lipid oxidation in muscles from cull-cows.
• This dataset is useful for meat science researchers to provide an overall view of the quality of stored meat combining global lipid content, lipid oxidation and color attributes.
• This dataset is useful for animal behavior scientists to study the effects of pre-slaughter stress on muscles of dairy cows and implications on stored meat qualities.
• This dataset is useful for the Lehning Laboratoires Company to promote the nutritional value of the patented diet supplement (PERP) for cattle.
• This dataset is useful for animal nutrition companies to investigate further the benefit of ingredients from the used diet supplementations (PERP and vitamin E).
• These data can be combined with data from other ruminant experiments in order to perform new and larger analyzes.
• These data can be used by statisticians and/or bioinformaticians to develop prediction models for meat quality.

## 1. Data description

The dataset, available without restriction at https://doi.org/10.15454/T6AMBC (portail DATA INRAE), reports raw data on muscle attributes and meat quality indicators from cull-cows. The list of meat quality indicators is detailed in the Table 1 included in this article. In Table 1, muscle at slaughter (D0) indicators are lipid contents (g/100 g of fresh tissue), fatty acid composition (% of total Fatty Acid Methyl Esters (FAME)), antioxidant enzyme activities, antioxidative status and indicators of overall lipid oxidation describing the lipid and antioxidative attributes of muscles. The meat (after 12 d of muscle aging and storage) indicators are antioxidative status, global lipid oxidation and surface color reflecting the nutritional and sensorial meat qualities. The treatments were diet supplementations, pre-slaughter stress (details can be found in [2]), type of aging and packaging storage conditions.

**Table 1:** Indicators of lipid status and color attributes in meat from slaughter to storage.

Indicators	Abbreviations (Unit)	Biological meanings	Methods	Technologies	Equipment	References
**Indicators of lipids content and fatty acids (FA) composition**						
Total lipids	Lipids (% fresh tissue)	Beef total lipid	*Folch*	Gravimetric method – direct extraction solvent		[3]
Tetradecanoic	14:0 (% of total FAME)	Centesimal FA composition % of total FAME of total lipids in tissue		Gas chromatography – flame ionization	Model chromatography – Perichrom 2100 – Périchrom, Saulx-les-Chartreux, France)	[4]
Hexadecanoic	16:0 (% of total FAME)	Centesimal FA composition % of total FAME of total lipids in tissue		Gas chromatography – flame ionization	Model chromatography – Perichrom 2100 – Périchrom, Saulx-les-Chartreux, France)	[4]
Octadecanoic	18:0 (% of total FAME)	Centesimal FA composition % of total FAME of total lipids in tissue		Gas chromatography – flame ionization	Model chromatography – Perichrom 2100 – Périchrom, Saulx-les-Chartreux, France)	[4]
Cis-9-octadecenoic + cis-11- octadecenoic	Sum 18:1 Δ9 cis + 18:1 Δ11 cis (% of total FAME)	Centesimal FA composition % of total FAME of total lipids in tissue		Gas chromatography – flame ionization	Model chromatography – Perichrom 2100 – Périchrom, Saulx-les-Chartreux, France)	[4]
Tras-9-octadecenoic + trans-11-octadecenoic	Sum 18:1 Δ9 trans + 18:1 Δ11 trans (% of total FAME)	Centesimal FA composition % of total FAME of total lipids in tissue		Gas chromatography – flame ionization	Model chromatography – Perichrom 2100 – Périchrom, Saulx-les-Chartreux, France)	[4]
9,12-octadecadienoic	18:2 n-6 (LA) (% of total FAME)	Centesimal FA composition % of total FAME of total lipids in tissue		Gas chromatography – flame ionization	Model chromatography – Perichrom 2100 – Périchrom, Saulx-les-Chartreux, France)	[4]
9,12,15-octadecatrienoic	18:3 n-3 (ALA) (% of total FAME)	Centesimal FA composition % of total FAME of total lipids in tissue		Gas chromatography – flame ionization	Model chromatography – Perichrom 2100 – Périchrom, Saulx-les-Chartreux, France)	[4]
5,8,11,14-eicosatetraenoic	20:4 n-6 (AA) (% of total FAME)	Centesimal FA composition % of total FAME of total lipids in tissue		Gas chromatography – flame ionization	Model chromatography – Perichrom 2100 – Périchrom, Saulx-les-Chartreux, France)	[4]
5,8,11,14,17-eicosapentaenoic	20:5 n-3 (EPA) (% of total FAME)	Centesimal FA composition % of total FAME of total lipids in tissue		Gas chromatography – flame ionization	Model chromatography – Perichrom 2100 – Périchrom, Saulx-les-Chartreux, France)	[4]
7,10,13,16,19-docosapentaenoic	22:5 n-3 (DPA) (% of total FAME)	Centesimal FA composition % of total FAME of total lipids in tissue		Gas chromatography – flame ionization	Model chromatography – Perichrom 2100 – Périchrom, Saulx-les-Chartreux, France)	[4]
4,7,10,13,16,19-docosahexaenoic	22:6 n-3 (DHA) (% of total FAME)	Centesimal FA composition % of total FAME of total lipids in tissue		Gas chromatography – flame ionization	Model chromatography – Perichrom 2100 – Périchrom, Saulx-les-Chartreux, France)	[4]
Sum FA CLA	Sum CLA (% Total FAME)	Sum				
Sum FA Total SFA	Total SFA (% Total FAME)	Sum				
Sum FA Total n-3 PUFA	Total n-3 PUFA (% Total FAME)	Sum				
Sum FA Total n-6 PUFA	Total n-6 PUFA (% Total FAME)	Sum				
Ratio n-6 / n-3	n-6/n-3	Ratio				
Ratio 18:2n-6 / 18:3n-3	18:2 n-6 / 18:3 n-3	Ratio				
Ratio PUFA / SFA	PUFA/SFA	Ratio				
Cis-9-octadecenoic	18:1 Δ9 cis (% of total FAME)	Centesimal FA composition % of total FAME of total lipids in tissue		Gas chromatography – flame ionization	Model chromatography – Perichrom 2100 – Périchrom, Saulx-les-Chartreux, France)	[4]
Sum FA Total *cis* MUFA	Total cis MUFA (% Total FAME)	Sum				
Sum FA Total *trans* MUFA	Total trans MUFA (% Total FAME)	Sum				
**Indicators of antioxidative status**						
Total antioxidant status	TAS (µmol TEAC/g tissue)	Antioxidant capacity determined comparatively to "trolox equivalent antioxidant capacity" (TEAC)	*Ex vivo*	Spectrophotometry	Uvikon XS	[5] adapted by [6]
Vitamin A	Vit A (µg/g tissue)	Lipophilic antioxidant	*Ex vivo*	High performance liquid chromatography	HPLC Kontron Sys1 – detector UV/Vis	[7]
Vitamin E	Vit E (µg/g tissue)	Lipophilic antioxidant	*Ex vivo*	High performance liquid chromatography – detector UV/Vis	HPLC Kontron Sys1 – detector UV/Vis	[5] adapted by [6]
Catalase activity	Catalase (µmol of degraded H_2_O_2_/min/mg protein)	Antioxidant enzyme	*Ex vivo*	Spectrophotometry	Uvikon double-beam XS	[8] adapted by [9]
Superoxide Dismutase activity	SOD (IU/mg protein)	Antioxidant enzyme	*Ex vivo*	Spectrophotometry	Uvikon double-beam XS	[10] adapted by [9]
Glutathion Peroxidase activity	GPx (µmol NADPH/min/mg protein	Antioxidant enzyme	*Ex vivo*	Spectrophotometry	Uvikon double-beam XS	[11]
**Indicators of global lipid oxidation**						
Malondialdehyde	MDA (µg/g Tissue)	End-product of PUFAs (bearing more than 2 unsaturations) oxidation	*Ex vivo*	High performance liquid chromatography – fluorescence detector	HPLC Perkin – Serie 200 – Fluorescence detector	[12]
**Indicators of surface color**						
Lightness	L*	Color coordinate	*Ex vivo*	Spectrophotometry	Uvikon 933	CIE 1976 L*a*b* color space
Redness	a*	Color coordinate	*Ex vivo*	Spectrophotometry	Uvikon 933	CIE 1976 L*a*b* color space
Yellowness	b*	Color coordinate	*Ex vivo*	Spectrophotometry	Uvikon 933	CIE 1976 L*a*b* color space
Oxygenation index	Indox (%)	Relative percentage of oxymyoglobine to total amount of myoglobin	*Ex vivo*	Spectrophotometry	Uvikon 933	[13]
Metmyoglobin	Met (%)	Relative percentage to total amount of myoglobin	*Ex vivo*	Spectrophotometry	Uvikon 933	[13]

FAME = Fatty Acid Methyl Esters.

CLA = Conjugated Linoleic Acid = c9,t11-18:2 + t10,c12-18:2 + cla cis + cla trans.

SFA = Saturated Fatty Acids.

LA = Linoleic Acid.

ALA = Alpha Linoleic Acid.

EPA = EicosaPentaenoic Acid.

DPA = DocosaPentaenoic Acid.

DHA = DocosaHexaenoic Acid.

MUFA = MonoUnsaturated Fatty Acid; PUFA = PolyUnsaturated Fatty Acids.

Total SFA = 12:0 + 14:0 + 16:0 + 18:0 + 20:0 + 21:0 + 22:0 + 23:0 + 24:0.

Total n-6 PUFA = 18:2n-6 trans, trans + 18:2n-6 trans,cis + 18:2n-6 cis,trans + 18:2n-6 cis,cis + 18:3n-6 + 20:2n-6 + 20:3n-6 + 20:4n-6 + 22:2n-6 + 22:4n-6 + 22:5n-6.

Total n-3 PUFA = 18:3n-3 + 20:3n-3 + 20:4n-3 + 20:5n-3 + 22:3n-3 + 22:4n-3 + 22:5n-3 + 22:6n-3.

Total PUFA = Total n-6 PUFA + total n-3 PUFA + total conjugated linoleic acid + total conjugated PUFA.

**Fig. 1:**
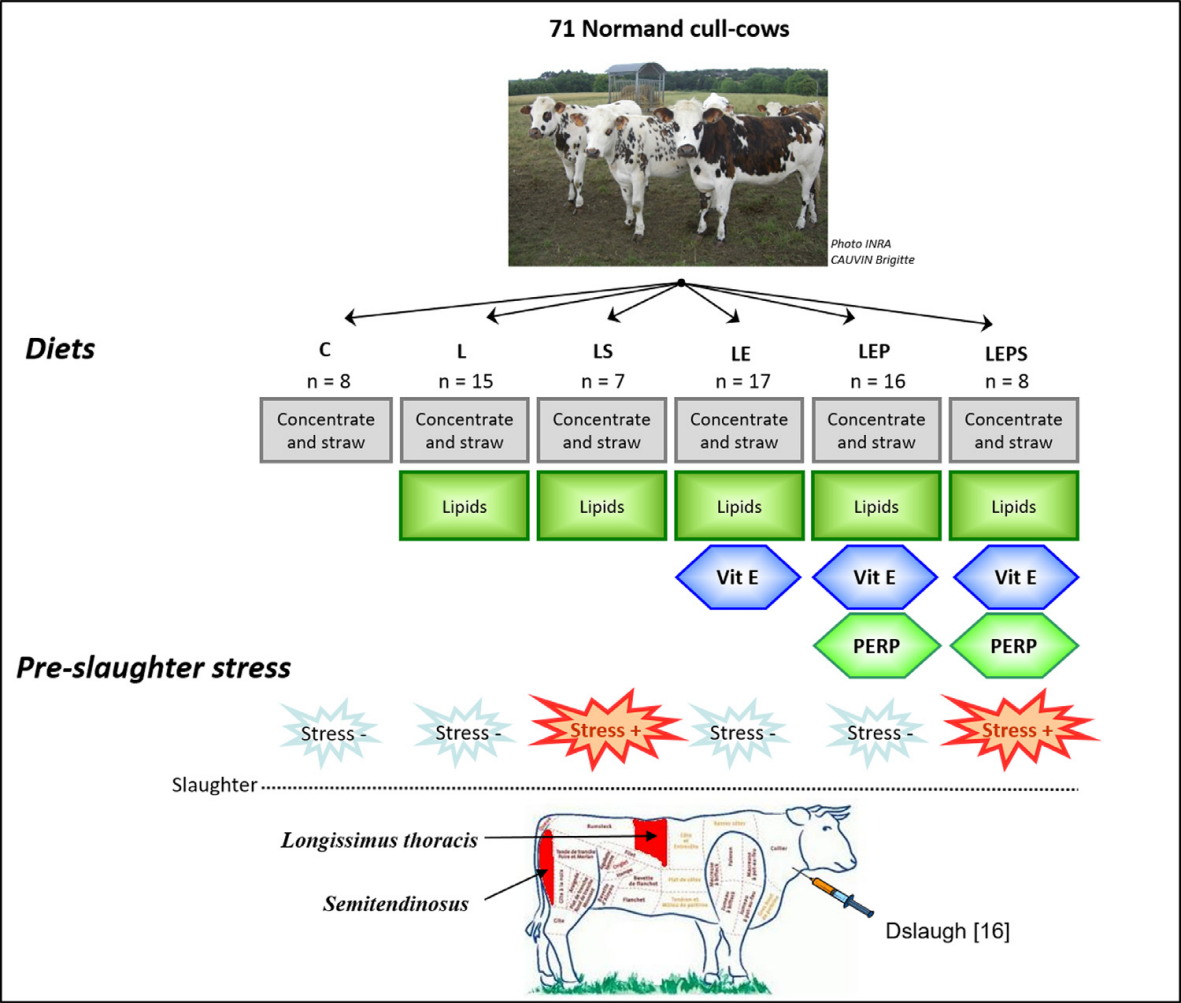
Experimentation designed to study the effects of 3 diet supplementations (lipids; lipids and vitamin E; lipids, vitamin E and PERP) and 2 slaughter protocols relative to pre-slaughter stress conditions on *Longissimus dorsi* and *Semitendinosus muscles* of Normand cull-cows at slaughter after a 100 days finishing period. Data on plasma at slaughter (Dslaugh) are reported in [14].

**Fig. 2:**
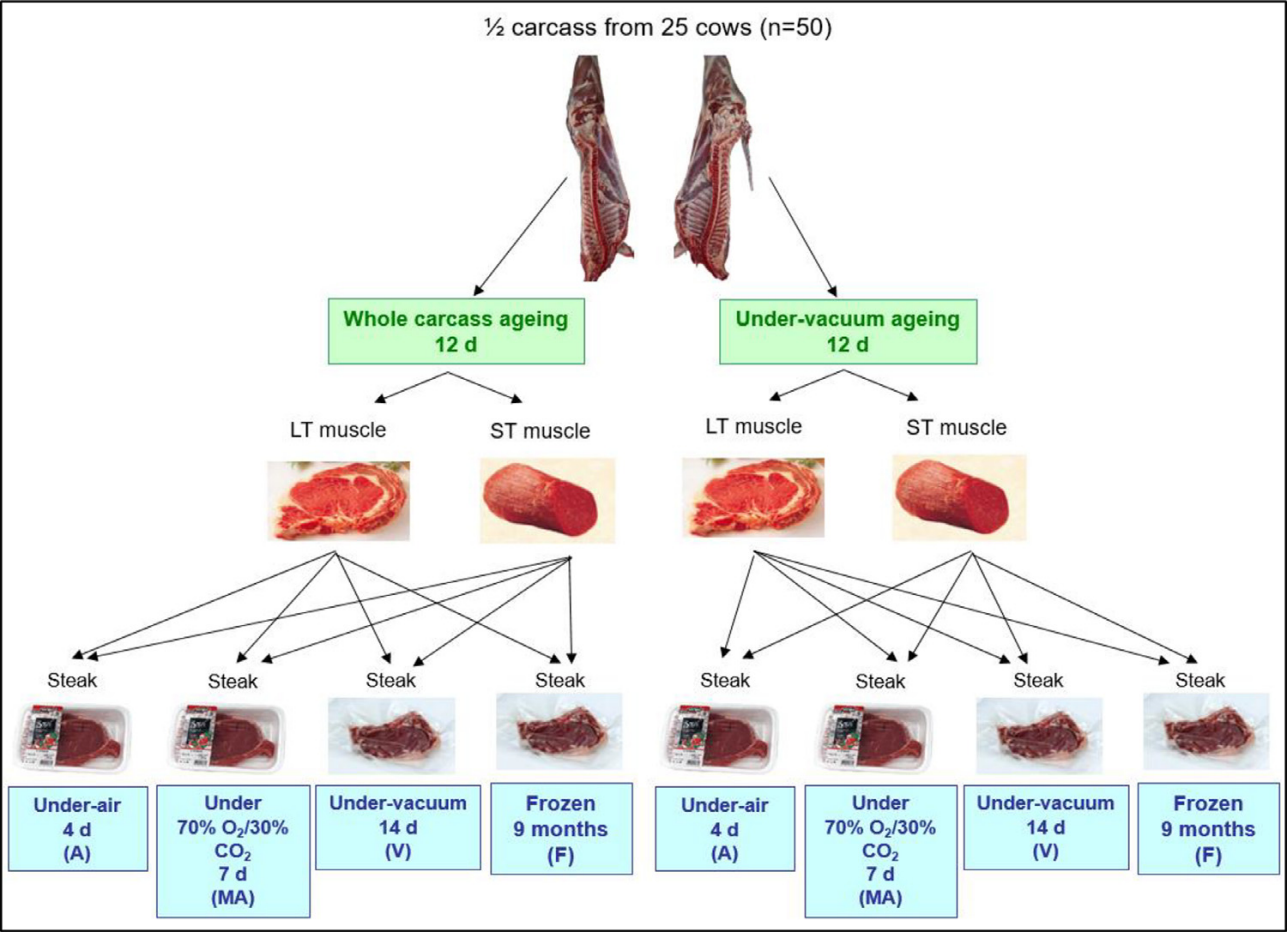
Experimentation designed to study the effects of two types of aging and four packaging storage conditions on *Longissimus dorsi* (LT) and *Semitendinosus* (ST) muscles from Normand cull-cows.

This data article reports illustrations of the experimental design with Fig. 1 referring to the animal breeding period, and Fig. 2 referring to the muscle and meat treatment after slaughter, both included in the “Animals” and “Muscle processing” sections within this article.

## 2 Experimental design, materials and methods

### 2.1 Animals

Seventy-five Normand cull cows, 48–60 months old with a mean live weight of 642 kg were used for this experiment led at Herbipole (Herbipole, INRAE, 2018. Low mountain ruminant experimental facility, doi:10.15454/1.5572318050509348E12). Three groups of 25 +/− 1 cows were conducted in winter 2007 (*n* = 24), summer and autumn 2007 (*n* = 25) and winter 2008 (*n* = 26). Numbers between 251 and 331 identify cows. Two cows presenting health problems during the finishing period (sarcosporidiose and abomasum flipping) were removed from the experimentation. Our other datapaper [14] on the same animals and project containing information on plasma during rearing and before slaughter contains two more cows that were discarded from the slaughter study for technical reasons (they were spare healthy cows), leaving 71 cows in the present paper.

### 2.2 Dietary treatments

During 101 ± 3 days, the 71 cows were fed a straw (30%) and concentrate (70%)-based diet (Fig. 1). Eight cows received no supplementation (Control group, **C**). For 63 of the 71 cows, the diet was supplemented with lipids (40 g oil/kg diet DM) provided by extruded oilseeds. Among these 63, 22 received no further supplement (**L** and **LS** groups), 17 cows received a diet supplemented with vitamin E only (155 IU/kg) (**LE** group) and 24 cows received a supplement of vitamin E (155 UI/kg) and plant extracts rich in polyphenols (PERP; 7 g/kg diet DM) (**LEP** and **LEPS** groups; Fig. 1). The PERP were prepared from rosemary (*Rosemarinus officinalis*), grape (*Vinis vitifera*), citrus (*Citrus paradisi*) and marigold (*Calendula officinalis*) by the Lehning Laboratoires company (Sainte-Barbe, France) (INRA patent #P170-B-23.495 FR). Pens were equipped with electronic feeding gates and individually offered the appropriate allowance of concentrates and straw per day for each cow. The experimental composition of the diets is reported in [14].

### 2.3 Pre-slaughter treatments

Two slaughter conditions were used at the end of the finishing period, for part of the treatments, limited stress *vs* moderate stress (stress − and stress + detailed in [14,15]). Hence, 7 of the 22 cows receiving no further dietary supplement, were slaughtered under moderate (LS) and 15 under limited stress conditions (L). Of the 24 cows receiving a supplement of vitamin E and PERP), 8 were slaughtered under moderate (LEPS) and 16 under limited stress conditions (LEP). The cows of the other treatments (C, L, LE and LEP) were all slaughtered using the limited stress conditions. Slaughters were led in Herbipole (Herbipole, INRAE, 2018. Low mountain ruminant experimental facility, doi:10.15454/1.5572318050509348E12).

### 2.4 Muscle processing

Carcasses were refrigerated at 4 °C for 24 h. Only 5 carcasses per group were processed, but carcasses from C group were not processed due to financial constraints. *Longissimus thoracis* (**LT**) and *semitendinosus* (**ST**) muscles from the right half carcass were removed, vacuum packed and refrigerated at 4 °C for 12 d (**under-vacuum aging**). The left half carcass was refrigerated at 4 °C for 12 d and LT and ST muscles were subsequently removed (**whole-carcass aging**). After aging, both muscles cut into 10–15 mm (LT) and 8–10 mm (ST) thick steaks of the type commonly found on the French market. All samples stored at 4 °C under a standard supermarket fluorescent light. Samples placed in an expanded polystyrene (PSE) tray type 049405 (Boulegon-Parry, France) overwrapped in a vinyl stretchable film 9 µm thick (Soussana, France) under air for 4 d (**A**). Samples placed in an polystyrene (EVOH) tray type 2450 (Form'plast, France) and packed under a modified atmosphere containing 70% O_2_/30% CO_2_, with a Multivac T200 using OPP-T504 AF / 20/30 film 52 µm thick (Soussana, France) for 7 d (**MAP**). The packaging gas provided by Linde-gas (France). Samples placed under vacuum with a Multivac C400 in a bag type 102353 (Boulegon-parry, France) for 14 d (**V**) and other vacuum packed samples frozen at −20 °C for 9 months (**F**) (Fig. 2). After the storage periods and immediately after opening the pack, each sample ground into a fine homogenous powder in liquid N_2_ and then stored at −80 °C until analysis.

### 2.5 Sample collection

Biochemical measurements were obtained from the LT and ST muscles at slaughter (D0), and from LT and ST meat obtained after whole-carcass or under-vacuum aging (D12). Finally, measurements were made on LT and ST meat, following aging during 4 d under-air (A), 7 d under 70% O_2_/30% CO_2_ (MAP), 14 d under vacuum (V) and 9 months under frozen (F) conditions.

The reported data are individual values that we want to open to the scientific community for a free re-use. Some meaning values were published in different original articles dealing with ruminant nutrition and/or meat quality [2,^16^,^17^,^18^,1].

## Appendix Supplementary materials

Image, application 1

## References

[1] Delosière M., Durand D., Bourguet C., Terlouw E.M.C. (2020). Lipid oxidation, pre-slaughter animal stress and meat packaging: can dietary supplementation of vitamin E and plant extracts come to the rescue?. Food Chem..

[2] Gobert M., Gruffat D., Habeanu M., Parafita E., Bauchart D., Durand D. (2010). Plant extracts combined with vitamin E in PUFA-rich diets of cull cows protect processed beef against lipid oxidation. Meat Sci..

[3] Folch J., Lees M., Sloane Stanley G.H. (1957). A simple method for the isolation and purification of total lipides from animal tissues. J. Biol. Chem..

[4] Bauchart D., Durand D., Scislowski V., Chilliard Y., Gruffat D., Hocquette J-F., Gigli S. (2005).

[5] Miller N.J., Rice-Evans C., Davies M.J., Gopinathan V., Milner A. (1993). A novel method for measuring antioxidant capacity and its application to monitoring the antioxidant status in premature neonates. Clin. Sci..

[6] Scislowski V., Bauchart D., Gruffat D., Laplaud P.M., Durand D. (2005). Effects of dietary n-6 or n-3 polyunsaturated fatty acids protected or not against ruminal hydrogenation on plasma lipids and their susceptibility to peroxidation in fattening steers. J. Anim. Sci..

[7] Hatam L.J., Kayden H.J. (1979). A high-performance liquid chromatographic method for the determination of tocopherol in plasma and cellular elements of the blood. J. Lipid Res..

[8] Aebi H. (1974).

[9] Gladine C., Morand C., Rock E., Gruffat D., Bauchart D., Durand D. (2007). The antioxidative effect of plant extracts rich in polyphenols differs between liver and muscle tissues in rats fed n-3 PUFA rich diets. Anim. Feed Sci. Technol..

[10] Marklund S., Marklund G. (1974). Involvement of superoxide anion radical in autoxidation of pyrogallol and a convenient assay for superoxide-dismutase. Eur. J. Biochem..

[11] Agergaard N., Jensen P.T. (1982). Procedure for blood glutathion-peroxidase determination in cattle and swine. Acta Vet. Scand..

[12] Agarwal R., Chase S.D. (2002). Rapid, fluorimetric-liquid chromatographic determination of malondialdehyde in biological samples. J. Chromatogr. B Analyt. Technol. Biomed. Life Sci..

[13] Krzywicki K. (1979). Assessment of relative content of myoglobin, oxymyoglobin and metmyoglobin at the surface of beef. Meat Sci..

[14] Delosière M., Thomas A., Terlouw C., Durand D. (2018). Plasma indicators of bovine health: impacts of diet supplementations and pre-slaughter stress. Data Brief..

[15] Bourguet C., Deiss V., Gobert M., Durand D., Boissy A., Terlouw E.M.C. (2010). Characterising the emotional reactivity of cows to understand and predict their stress reactions to the slaughter procedure. Appl. Anim. Behav. Sci..

[16] Habeanu M., Thomas A., Bispo E., Gobert M., Gruffat D., Durand D (2014). Extruded linseed and rapeseed both influenced fatty acid composition of total lipids and their polar and neutral fractions in longissimus thoracis and semitendinosus muscles of finishing Normand cows. Meat Sci..

[17] Delosière M., Parafita E., Habeanu M., Gruffat D., Durand D. (2018). Dietary plant extracts combined with vitamin e limit the discoloration in stored n-3 PUFA rich meat. Agric. Sci..

[18] M. Gobert, D. Bauchart, E. Parafita, D. Durand, in *57th International Congress of Meat Science and Technology*, 2011, 109–112 (Meat Science).

